# Mono-(2-ethylhexyl) phthalate Promotes Dengue Virus Infection by Decreasing IL-23-Mediated Antiviral Responses

**DOI:** 10.3389/fimmu.2021.599345

**Published:** 2021-02-15

**Authors:** Chun-Yu Lin, Chung-Hao Huang, Wen-Hung Wang, Jyrki Tenhunen, Ling-Chien Hung, Chi-Chou Lin, Yu-Cheng Chen, Yen-Hsu Chen, Wei-Ting Liao

**Affiliations:** ^1^ Division of Infectious Diseases, Department of Internal Medicine, Kaohsiung Medical University Hospital, Kaohsiung Medical University, Kaohsiung, Taiwan; ^2^ School of Medicine, Graduate Institute of Medicine, College of Medicine, Center for Tropical Medicine and Infectious Diseases Research, Kaohsiung Medical University, Kaohsiung, Taiwan; ^3^ Department of Surgical Sciences, Department of Medical Biochemistry and Microbiology, Uppsala University, Uppsala, Sweden; ^4^ Department of Biotechnology, College of Biomedical Science, Kaohsiung Medical University, Kaohsiung, Taiwan; ^5^ Department of Internal Medicine, Kaohsiung Municipal Ta-Tung Hospital, Kaohsiung, Taiwan; ^6^ Department of Biological Science and Technology, College of Biological Science and Technology, National Chiao Tung University, HsinChu, Taiwan; ^7^ Department of Medical Research, Kaohsiung Medical University Hospital, Kaohsiung Medical University, Kaohsiung, Taiwan; ^8^ Research Center for Environmental Medicine, Kaohsiung Medical University, Kaohsiung, Taiwan

**Keywords:** phthalate, dengue virus, macrophage, IL-23, biological sciences

## Abstract

Exposure to environmental hormones such as di(2-ethylhexyl) phthalate (DEHP) has become a critical human health issue globally. This study aimed to investigate the correlations between DEHP/mono-(2-ethylhexyl) phthalate (MEHP) levels and macrophage-associated immune responses and clinical manifestations in dengue virus (DV)-infected patients. Among 89 DV-infected patients, those with DV infection-related gastrointestinal (GI) bleeding (n = 13, 15% of patients) had significantly higher DEHP exposure than those without GI bleeding (n = 76, 85% of patients), which were 114.2 ng/ml versus 52.5 ng/ml ΣDEHP in urine; p = 0.023). In an *in vitro* study using cultured human monocyte-derived macrophages (MDMs) to investigate the effects of MEHP, treatment increased IL-1β and TNF-α release but decreased IL-23 release, with negative correlations observed between urine ΣDEHP and serum IL-23 levels in patients. MEHP-treated MDMs had lower antiviral Th17 response induction activity in mixed T-cell response tests. The *in vitro* data showed that MEHP increased DV viral load and decreased IL-23 release dose-dependently, and adding IL-23 to MEHP-exposed MDMs significantly reduced the DV viral load. MEHP also suppressed IL-23 expression *via* the peroxisome proliferator-activated receptor-gamma (PPAR-γ) pathway. Further, the PPAR-γ antagonist GW9662 significantly reversed MEHP-induced IL-23 suppression and reduced the DV viral load. These study findings help to explain the associations between high MEHP levels and the high global burden of dengue disease.

## Introduction

Dengue fever is caused by dengue virus (DV) infection mediated by a mosquito (*Aedes aegypti* or *Aedes albopictus*) bite and occurs predominantly in tropical and subtropical areas. The World Health Organization (WHO) reports that the prevalence of dengue fever has increased continuously for nearly 30 years in more than 100 countries (1). The clinical manifestations of DV infection might include asymptomatic presentation, mild fever, typical dengue rash, severe dengue hemorrhagic fever (DHF), and dengue shock syndrome (DSS). The WHO estimates that 2.5 billion people are at risk of DV infection, and 390 million people are infected (2). Among those infected, 96 million show clinical manifestations, and 0.5 million are identified as having DHF (3).

Along with global climate change, DV infection has become a major threatening to public health all around the world (4). In 2014, a severe outbreak (> 15,000 cases) of dengue fever occurred in southern Taiwan after an illegal di(2-ethylhexyl) phthalate (DEHP) food additive incident that affected a large percentage of the national population (5). During the outbreak, more than 120 DHF cases and nearly 20 deaths were reported at Kaohsiung Medical University Hospital. Since the safety of a dengue vaccine for humans is currently controversial (6), it is imperative to clarify the risk factors associated with severe DV infection. Phthalate exposure has been reported to alter macrophage functions (7) and, therefore, probably contributes to the development of an increasing number of DV-related infectious complications (8).

DEHP is a compound used in large quantities in polyvinyl chloride (PVC) processing industries to increase the flexibility of PVC plastics. Therefore, DEHP exposure is a global environmental problem that occurs commonly *via* the daily usage of plastic food/drink containers and plastics used in medical equipment. The estimated average human DEHP intake in the general population is 0.71–4.6 µg/kg/day (9). However, among hemodialysis patients, exposure might average 75.2 mg of DEHP during a single dialysis session (10). After exposure, DEHP rapidly converts to the phthalate metabolite mono-(2-ethylhexyl) phthalate (MEHP) in the human body *via* the phase I xenobiotic metabolic process in the liver (11–13). MEHP alters macrophage functions, including increased TNF-α and prostaglandin secretion (7, 14).

Primary human splenic macrophages are the major target cells for DV (15, 16). Surface receptors on macrophages are required for DV infection, including ICAM3-grabbing non-integrin receptors, DC-SIGN, L-SIGN, high-affinity laminin receptor, mannose receptor, and glucose-regulated protein 78 (GRP78). After infection is established, the macrophages release many cytokines, including IFN-γ, TNF-α, IL-8, IL-6, IL-1β, and IL-23, which leads to different pathologic outcomes (17–19). For example, TNF-α and IL-8 increase the permeability of the vessel endothelial cells, resulting in vascular leakage and shock (DHF/DSS) (20). In contrast, IL-23 released from DV-infected macrophages initiates antiviral responses by activating Th17-associated responses (21).

Therefore, the present study aimed to determine the correlations, as well as underlying mechanisms, between phthalate compound exposure and DV infection using IRB-proven (KMUH-IRB-20140303) samples from the 2014 DV outbreak. We hypothesized that since MEHP might alter macrophage cytokine expression and responses, it could also change the outcomes of DV infection. Clinical investigations were conducted on DV-infected patients and on MEHP-exposed human monocyte-derived macrophages (MDMs) to test this hypothesis and investigate the biological effects of MEHP exposure on macrophages. Since plastic items are used globally, and phthalate contamination is almost unavoidable, understanding the interactions between MEHP and DV-infection might provide new evidence-based insights into this modern health issue.

## Materials and Methods

### Ethics Statement

The human study protocol of the present study was approved by the Institutional Review Board (IRB) of Kaohsiung Medical University Hospital (IRB Number: KMUH-IRB-20140303). Signed informed consent was obtained from all subjects.

### Patient Enrollment

Patients aged ≥20 years were enrolled prospectively, and urine samples (to measure DEHP exposure; see the following section) were provided on the first day of hospital consultation. The time of symptom onset for each patient was recorded immediately after enrollment to minimize recall bias. Patients who visited the hospital more than 6 days after symptom onset were excluded. All patients were diagnosed with dengue fever, and all methods used for the management of the enrolled patients were in accordance with the 2009 WHO guidelines (1). WBC counts and platelet counts from peripheral blood samples were measured at least every other day until patient recovery. Patients’ demographic and clinical data, including preexisting concomitant diseases, clinical manifestations, and laboratory data, were collected using standardized data collection forms.

### Diagnosis of DV Infection

All laboratory diagnostic tests for the identification of DV infection were performed at laboratories accredited by the Centers for Disease Control, Department of Health, Taiwan. The diagnosis of dengue fever was confirmed by at least one positive result of real-time polymerase chain reaction (RT-PCR) or a positive test for dengue nonstructural protein 1 (NS1) Ag STRIP (Bio-Rad Laboratories, Marnes-la-Coquette, France) (4). Dengue viral RNA from patient serum was extracted using a Viral RNA mini kit (Qiagen, Cat. No. 52906, Chatsworth, CA, USA) according to the manufacturer’s instructions. All dengue viral RNAs were treated with RQ1 RNase-free DNase I before being used as templates for cDNA synthesis. For reverse transcription, the PrimeScript reagent kit (TaKaRa, Cat. No. RR037A, Kyoto, Japan) was used according to the manufacturer’s instructions. The resulting cDNA was evaluated for dengue viral serotypes by RT-PCR (Applied Biosystems, Foster 7500, CA, USA). The primers and probes used are listed in **Supplementary Table S1**.

### Cytokine Profiles of Patients With Dengue

The serum levels of several cytokines were measured in some patients using commercial kits (cat no. 560484 and 552990, BD Biosciences, San Jose, CA, USA), following the manufacturer’s instructions, including IL-1β, IL-18, IL-23, IL-17, INF-γ, TNF-α, IL-10, IL-6, IL-4, IL-2, IP-10, MCP-1, MIG, RANTES, and IL-8.

### Measurement of DEHP Exposure in Urine Samples

Urine samples from all patients were collected in polypropylene containers after enrollment and then transferred to an acetonitrile-prewashed amber glass bottle. The samples were stored at −20°C until analysis. Phthalate metabolites were analyzed using column-switching high-performance liquid chromatography tandem mass spectrometry (LC-MS/MS) (22). In brief, this method starts with the digestion of proteins in the mixture; the resulting peptides are separated by liquid chromatography (LC) and identified by tandem mass spectrometry (MS). Equivalent DEHP exposure was measured as previously described (23). The summary DEHP (ΣDEHP) metabolite measurement was calculated based on the molar sum of MEHP, MEOHP, MEHHP, and MECPP, according to the following formula:

(weight concentration of MEHP/278.3)+(MEOHP/292.3)+(MEHHP/294.3)+(MECPP/308.3)]×390.6

(23).

### Human Monocyte-Derived Macrophages

The *in vitro* part of this study was approved by the Institutional Review Board of Kaohsiung Medical University (KMUH-IRB-20140303). Normal human primary CD14+ monocytes were isolated from peripheral blood mononuclear cells by magnetic bead sorting with an anti-CD14 monoclonal antibody (MACS, Miltenyi Biotec, Germany) according to the manufacturer’s instructions (24). The isolated CD14+ cells were first incubated in a culture medium with 100 ng/ml of recombinant human GM-CSF for 3 days, followed by stimulation with 10 ng/ml LPS and 50 ng/ml IFN-γ for 3 additional days to obtain MDMs. MDMs (10^6^/ml) were cultured in RPMI 1640 medium with 10% FBS at 37°C in a humidified incubator with 5% CO_2_ for NIR treatments.

### Enzyme-Linked Immunosorbent Assay

After MEHP treatment *in vitro*, cell-free supernatants from cultured MDMs were obtained to test the concentrations of cytokines by ELISA. Human IL-1β, IL-6, IL-8, TNF-α, IL-10, IL-12, IL-23, IFN-γ, IL-13, and IL-17 ELISA kits (R&D Systems, Minneapolis, MN, USA) were used according to the manufacturer’s instructions. Chemokine or cytokine concentrations were calculated based on linear regression standard curves, in which r^2^ was higher than 0.99.

### DV Infection and Quantification of the DV RNA

MDMs at a density of 1 × 10^5^ cells/ml were infected with DV at a multiplicity of infection of 0.1 for 2 hours. After the infection, the supernatant was removed, and the cells were incubated with various concentrations of MEHP for an additional 2 days.

For the quantification of DV RNA, total cellular RNA from treated MDMs was extracted using the Total RNA Miniprep Purification Kit (GMbiolab, Taiwan) according to the manufacturer’s instructions. The expression level of DV NS5 mRNA was determined by quantitative real-time RT-PCR (qRT-PCR) with specific primers corresponding to the DV NS5 gene as follows: forward primer, 5-AAG GTG AGA AGC AAT GCA GC-3, and reverse primer, 5-CCA CTC AGG GAG TTC TCT CT-3. The copy number of NS5 in each *in vitro* sample was normalized to the endogenous reference gene glyceraldehydes-3-phosphate dehydrogenase (GAPDH), with the following primers: forward primer, 5-GTC TTC ACC ATG GAG AA-3, and reverse primer, 5-ATG GCA TGG ACT GTG GTC AT-3. The CT value of each sample was determined using the ABI Step One Real-Time PCR System (ABI Warrington, UK).

### PPARγ Luciferase Activity Assay

A commercially obtained peroxisome proliferator-activated receptor-gamma (PPARγ) promoter firefly luciferase vector was used to detect PPARγ promoter activity (with *Renilla* luciferase as an internal control). Cells grown in 24-well plates were transfected with 1 μg LipofectAMINE 3000 reagent, 4 μl LipofectAMINE-Plus reagent (Invitrogen, Carlsbad, CA), 10 ng *Renilla* control vector, and 80 ng of PPARγ firefly vector (Clonetech, Palo Alto, CA). The cells were incubated in the aforementioned mixture for 6 h at 37°C. After incubation, the transfection mixture was exchanged with a culture medium with or without MEHP treatment. Luciferase activity was measured using the Dual-Luciferase Reporter Assay system (Promega, Madison, WI) according to the manufacturer’s protocol.

### Statistical Analysis

Categorical data are presented as numbers; the chi-square test or Fisher’s exact test was performed for categorical data. Univariate analyses were performed to determine which factors or manifestations were associated with high or low phthalate exposure. Continuous variables were analyzed by Student’s t-tests unless otherwise described. Statistical analyses were performed using IBM SPSS statistical software version 22 for Windows (IBM Corp., Armonk, New York, USA). A two-tailed p-value <0.05 was considered statistically significant. For *in vitro* experimental samples, a two-tailed Kruskal-Wallis test was used to calculate significance. All error bars in the graphs indicate the standard deviation (SD).

## Results

### DV-Infected Patients With High Urine ΣDEHP Concentrations Are at a Higher Risk of Chills and Gastrointestinal Bleeding

**Figure 1:**
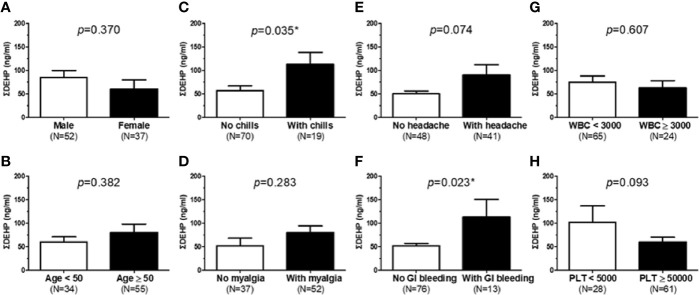
Analysis of correlations between DEHP exposure and typical symptoms in 89 dengue patients. **(A, B)** DEHP levels between sex and age were analyzed. **(C)** DEHP levels between dengue patients with or without chills. **(D)** DEHP levels between dengue patients with or without myalgia. **(E)** DEHP levels between dengue patients with or without headache. **(F)** DEHP levels between dengue patients with or without gastrointestinal (GI) tract bleeding. **(G)** DEHP levels between white blood cell (WBC) counts (cells/μl) in dengue patients. **(H)** DEHP levels between platelet (PLT) counts (cells/μl) in dengue patients. Patients with chills and GI bleeding showed significantly higher levels of DEHP. *P < 0.05 by Student’s t-test (means ± SD).

A total of 89 DV-infected patients in a medical center in Southern Taiwan were enrolled between August and December 2014. The patients’ demographic and clinical characteristics are shown in **Table 1**. Further analysis of dengue patients revealed typical dengue symptoms such as chills, headache, myalgia, and gastrointestinal (GI) bleeding. Patients with chills and GI bleeding showed significantly higher levels of DEHP exposure (without chills = 55.4 ng/ml ΣDEHP in urine versus chills = 114.3 ng/ml; p = 0.035; without GI bleeding = 52.5 ng/ml ΣDEHP in urine versus with GI bleeding = 114.2 ng/mL; p = 0.023; **Figure 1**). However, upon dividing patients into “low DEHP exposure” and “high DEHP exposure” groups according to the median value of ΣDEHP (35.3 ng/ml), a significantly higher proportion of DV patients in the high-exposure group experienced chills and GI bleeding, suggesting that DEHP exposure is correlated with more clinical complications, and at least DV infection-associated chills and GI bleeding.

**Table 1:** Demographic and clinical characteristics and laboratory data of patients with dengue virus infection.

	ΣDEHP			
	Low	High	Univariate	
	(*n*=45)	(*n*=44)	OR (95% CI)	*p* value
**Demographic data**				
Sex, male	23 (51.1)	29 (65.9)	1.9 (0.7-4.8)	0.230
Age ≥ 65 years	7 (15.6)	12 (27.3)	2.0 (0.7-5.8)	0.276
Median age (IQR)	51 (38-63)	56 (42-64)		0.173
Presentation time (median, day after onset)	1 (0-3)	1 (1-3)		0.318
**Pre-existing medical condition**				
Peptic ulcer disease	2 (4.4)	4 (9.1)	2.2 (0.4-12.3)	0.652
Hepatitis B virus	10 (22.2)	3 (6.8)	0.3 (0.1-1.0)	0.079
Hepatitis C virus	1 (2.2)	2 (4.5)	2.1 (0.2-23.8)	0.984
Hypertension	10 (22.2)	12 (27.3)	1.3 (0.5-3.4)	0.759
Diabetes mellitus	6 (13.3)	7 (15.9)	1.2 (0.4-4.0)	0.965
Chronic kidney disease	1 (2.2)	5 (11.4)	5.6 (0.6-49.8)	0.195
Malignancy, solid tumors	4 (8.9)	3 (6.8)	0.8 (0.2-3.6)	0.999
**Symptoms and signs at presentation**				
Fever > 38°C	45 (100)	44 (100)	not available	
Chills	4 (8.9)	15 (34.1)	5.3 (1.6-17,5)	0.008*
Headache	17 (37.8)	21 (47.7)	1.5 (0.6-3.8)	0.463
Retroorbital pain	4 (8.9)	5 (11.4)	1.3 (0.3-6.4)	0.739
Bone pain	6 (13.3)	14 (31.8)	3.0 (0.9-10.2)	0.067
Joints pain (arthralgia)	6 (13.3)	5 (11.4)	0.8 (0.2-3.4)	0.968
Myalgia	21 (46.7)	27 (61.4)	1.8 (0.7-4.6)	0.239
Skin rash	18 (40.0)	17 (38.6)	0.9 (0.4-2.4)	0.932
Petechiae	9 (20.0)	4 (9.1)	0.4 (0.1-1.6)	0.247
Abdominal pain	10 (22.2)	10 (22.7)	1.0 (0.3-3.1)	0.844
Nausea/vomiting	13 (28.9)	9 (20.5)	0.6 (0.2-1.9)	0.499
GI bleeding	2 (4.4)	11 (25.0)	7.1 (1.5-34.5)	0.014*
One or more WSs	17 (37.8)	17 (38.6)	1.0 (0.4-2.7)	0.893
**Laboratory data throughout the illness**				
Nadir PLT < 50,000/μl	11 (24.4)	16 (36.4)	1.8 (0.6-4.9)	0.321
Nadir WBC < 3,000/μl	28 (62.2)	29 (65.9)	1.2 (0.5-3.1)	0.887

IQR, interquartile range; GI, gastrointestinal; WSs, warning signs; PLT, platelet; WBC, white blood cell.

*p < 0.05, significantly different between the low and high ΣDEHP exposure groups. Bold values mean that these p values are < 0.05.

### MEHP Decreases IL-23 Expression in Cultured Human MDMs and DV-Infected Patients

**Figure 2:**
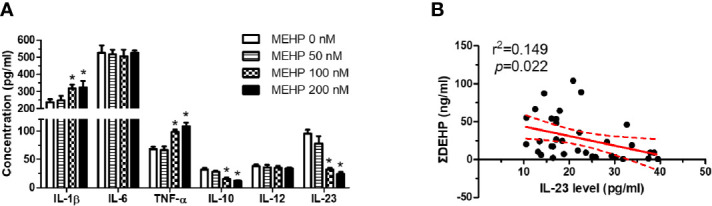
MEHP decreases IL-23 expression in cultured human MDMs. **(A)** MEHP altered inflammatory cytokines in cultured human monocyte-derived macrophages (MDMs). *P < 0.05 MEHP-treated vs. controls by Kruskal-Wallis test; N = 8, means ± SD. **(B)** Urine MEHP levels correlated negatively with serum IL-23 levels in dengue virus (DV)-infected patients. N = 43 by linear regression analysis.

We performed *in vitro* MEHP cytotoxicity screening (1 nM to 1 mM) using commercialized Cell Proliferation XTT Assay Kit (Roche, Germany). MEHP lower than 0.1 mM did not show cytotoxic effects, and the ID50 of MEHP in human MDM is about 0.5 mM (**Supplementary Figure S1**). We selected 50, 100, and 200 nM of MEHP as treatment dosages. Results of screening for MEHP-induced alterations in DV infection-associated macrophage cytokines/chemokines in cultured MDMs revealed that MEHP increased IL-1β and TNF-α release but decreased IL-10 and IL-23 release in human MDMs (**Figure 2A**). Among the 15 DV infection-associated cytokines in patient samples (**Supplementary Table S2–S6**), the decreased IL-23 level was validated in DV-infected patients with higher ΣDEHP exposure (**Figure 2B**). In DV-infected patients, the urine ΣDEHP concentrations were negatively correlated (p = 0.022) with serum IL-23. However, no correlations were found between ΣDEHP levels and other cytokines (IL-1β, TNF-α, and IL-10) in DV-infected patients.

### MEHP Decreases the Th17 Response in Cultured Human MDMs and DV-Infected Patients

**Figure 3:**
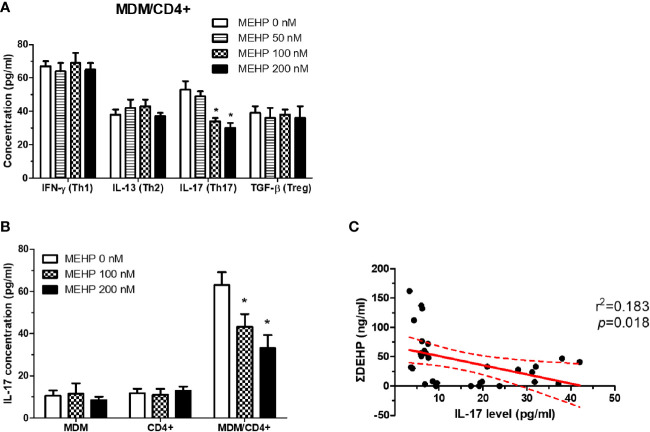
MEHP decreases the Th17 response in cultured human MDMs. **(A)** Cytokines released in supernatants from co-cultured monocyte-derived macrophage (MDM)/CD4+ cells with or without MEHP treatments were tested by ELISA. *P < 0.05 MEHP-treated vs. controls by Kruskal-Wallis test; N = 8, means ± SD. **(B)** IL-17 release in MDMs, CD4+ cells, and co-cultured MDM/CD4+ cells was tested by ELISA. *P < 0.05 MEHP-treated vs. controls by Kruskal-Wallis test; N = 3, means ± SD. **(C)** The urine MEHP level showed a negative correlation tendency with serum IL-17 levels in dengue virus (DV)-infected patients. N = 43 by linear regression analysis.

IL-23 promotes antiviral effects by activating Th17 cells. Results of mixed T-cell responses after MDM and CD4+ cell co-culture showed that IL-17 release was decreased in MEHP-treated MDM cells **(** **Figures 3A, B**). Decreased IL-17 was also observed in DV-infected patients for whom urine ΣDEHP concentrations correlated negatively with serum IL-17 levels (**Figure 3C**), suggesting that MEHP might alter antiviral responses, at least in part, by decreasing IL-23/IL-17-mediated mechanisms.

### MEHP Increases DV Infection in Human MDMs

**Figure 4:**
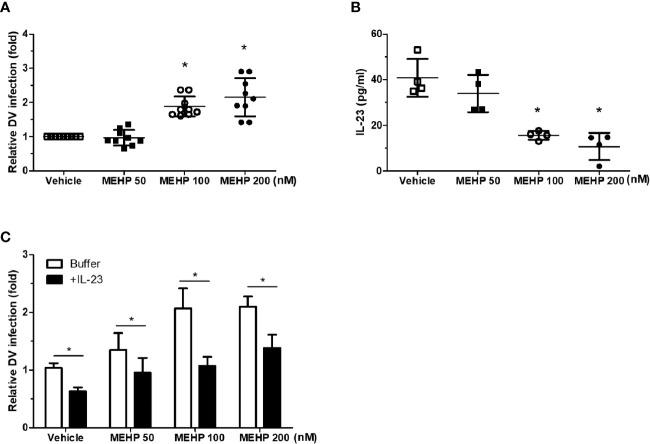
IL-23 treatments decrease dengue virus (DV) infection in cultured human MDMs. **(A)** Human monocyte-derived macrophages (MDMs) were infected by DV and the relative viral counts were detected by real-time PCR. *P < 0.05 by Kruskal-Wallis test; N = 9, means ± SD. **(B)** Human MDMs were infected by DV, and the IL-23 levels in supernatants were detected by ELISA. *P < 0.05 by Kruskal-Wallis test; N = 4, means ± SD. **(C)** DV-infected human MDMs were treated with 100 pg/ml of IL-23 and the relative viral counts were detected by real-time PCR. *P < 0.05 by Kruskal-Wallis test; N = 4, means ± SD.

Correlations between MEHP treatment and DV infection in MDMs revealed that MEHP treatment dose-dependently increased the viral load (**Figure 4A**), whereas IL-23 release from DV-infected MDMs was dose-dependently decreased (**Figure 4B**). Moreover, MEHP-induced DV viral load increases were significantly suppressed by applying 100 pg/ml of recombinant human IL-23 (**Figure 4C**), indicating that IL-23 contributes to antiviral responses in DV infection.

### MEHP Regulates IL-23 Expression *via* PPARγ

**Figure 5:**
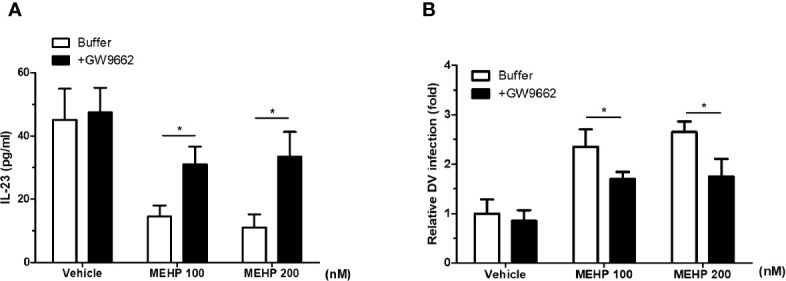
PPARγ is involved in MEHP-induced IL-23 downregulation. **(A)** GW9662 significantly increased IL-23 levels in MEHP-treated monocyte-derived macrophages (MDMs). **(C)** GW9662 significantly decreased DV viral loads in MEHP-treated MDMs. *P < 0.05 by Kruskal-Wallis test; N = 6, means ± SD.

IL-23 production was known to be inhibited by PPAR**γ** agonist (25). MEHP is known to have a U-shaped dose–response effect on PPARγ promoter activity (0.1 and 1 μM MEHP showed inhibitory effects on PPARγ, and 10 μM showed activation effects) (26). To elucidate whether MEHP poses a similar effect to the PPAR**γ** pathway on IL-23 regulation, we performed the following experiments. Using luciferase assays, MEHP treatment decreased PPARγ binding activity to the PPAR responding element (**Supplementary Figure S2**). In parallel, the PPARγ antagonist GW9662 (Sigma, St. Louis, MO) recovered the IL-23 levels and reduced the DV viral load after MEHP treatment (**Figures 5A, B**). These findings suggest that the PPARγ-modulated activity of MEHP is a key regulatory mechanism of IL-23-mediated anti-DV responses.

## Discussion

The findings of the present study provide clues about how phthalate might affect DV-infected patients, causing them to present with obvious or less obvious symptoms or even to remain asymptomatic. Recently, DV-infected patients without symptoms (i.e., asymptomatic infection) have attracted more attention because they could present with viremia but otherwise remain asymptomatic (18). Theoretically, these asymptomatic patients will not seek medical consultation but will still be a reservoir for DV transmission. Therefore, elucidating how DV-infected patients might present with non-dominant symptoms or even remain asymptomatic might help to avoid such transmission. In the present study, DV-infected patients who presented with more obvious symptoms, such as chills, also had significantly higher phthalate exposure levels. Patients with higher phthalate exposure were associated with lower circulating levels of IL-23. *In vitro*, MEHP was found to suppress IL-23 expression *via* the PPAR-γ pathway, resulting in increased viral infection.

The massive loss of blood is rare in vascular leakage and shock (DHF/DSS), and, when present, it is largely restricted to the gastrointestinal tract (27). In general, the presence of DV in several organs is not associated with gross or microscopic evidence of severe organ pathology, which agrees with the pathogenesis of DHF/DSS (27). Similar organ tropism has been observed in the primate model, with high concentrations of the virus isolated from the skin and gastrointestinal tract, whereas low concentrations of the virus were recovered from the spleen, thymus, and several peripheral lymph nodes (28). The authors of that study concluded that a high viral load in the blood and possibly viral tropism for endothelial cells, severe thrombocytopenia, and platelet dysfunction might result in characteristically increased capillary fragility, clinically manifested as petechiae, easy bruising, and GI mucosal bleeding. In a recent study that also included data from the 2014 Taiwan outbreak of DV-infection (4), NS1 and endothelial hyperpermeability were noted by us for their contribution to vascular leakage, which is characteristic of the disease.

In the present study, higher levels of DEHP exposure were observed in dengue patients with GI bleeding. TNF-α and IL-8 are known to increase the permeability of blood vessel endothelial cells, resulting in vascular leakage. Although the present study showed that MEHP treatment increased TNF-α release from MDMs, no correlations were found between circulating TNF-α and ΣDEHP. Recently, other animal studies demonstrated that DV NS1 triggers localized vascular leaks in the dorsal dermis of wild-type C57BL/6 mice by altering endothelial glycocalyx components, but this was found to be independent of inflammatory cytokines (TNF-α, IL-6, IL-8) (29, 30). Supernatants from DV-infected M1-macrophages (M1-Mϕ) are more potent than those from DV-infected M2-Mϕ in terms of increasing the permeability of endothelial cells (31). Therefore, further studies are required to fully understand the interactions between MEHP and endothelial permeability.

Human Mϕ and dendritic cells (DCs) are the primary targets of DV infections. Unlike DCs, which undergo apoptosis during DV infection, human Mϕ survive for at least 45 days after DV infection, suggesting that they might be regarded as a major source of proinflammatory cytokines *in vivo* (20, 32). However, whether human inflammatory macrophage subsets display distinct reactions to DV infection has not been addressed systematically. Based on mycobacterial infection studies, M1-Mϕ secrete high levels of IL-23 (p40/p19) but not IL-12 (p40/p35) after mycobacterial infection, whereas a secondary signal, IFN-γ, induces IL-12p35 transcription and IL-12 production. In contrast to M1-Mϕ, M2-Mϕ predominantly produce IL-10 but not IL-12 and IL-23. In addition, only M1-Mϕ, and not M2-Mϕ, support the Th1 response after mycobacterial infection. These results indicate that IL-23, but not IL-12, is the major type 1 cytokine produced by mycobacteria-stimulated M1-Mϕ (33–35).

Prominent examples show that the M protein of DV directly induces cell death in infected macrophages, and bystander cells block acute antiviral responses, contribute to local tissue damage, and attenuate the efficient progression of macrophage polarization toward an M1 phenotype. All of these processes, in turn, contribute to compromised antiviral immunity, leading to a high incidence of mortality or chronic viral persistence. The stimulation of TLRs in M1-Mϕ with DV can induce the activation of NF-κB and MAPK, which promotes the transcription of a range of proinflammatory cytokines (20). NLRP3 inflammasome-activated caspase-1 further processes pro-IL-1β and pro-IL-18 into their mature cytokine forms, IL-1β and IL-18. IL-1β can also enhance the production of IL-23 and IL-6. The released IL-1β, IL-18, and IL-23 induce Th17/γδ T cells to produce proinflammatory cytokines, which are responsible for host immune responses against DV infection (18, 20). Data from the present study show that MEHP suppresses the IL-23/IL-17 axis by interacting with PPAR-γ; this is because IL-23/IL-17 plays a significant role in host immunity against DV infection. These data also show that MEHP exposure increases DV viral load. Therefore, our data suggest that MEHP functions as an inhibitory agent against host immunity during DV infection, which might lead to more severe clinical manifestations such as GI bleeding.

DEHP exposure is a contemporary global environmental issue, and, in parallel with global warming, the prevalence of DV infection continues to increase. The findings of the present study provide evidence that the DEHP metabolite MEHP alters host immunity, at least in part, during DV infection, providing new insights into the correlation between DEHP exposure and more severe outcomes of DV infection. The results of this study might provide a useful academic reference for human health in the face of these two contemporary environmental issues.

## Data Availability Statement

The original contributions presented in the study are included in the article/**Supplementary Material**. Further inquiries can be directed to the corresponding authors.

## Ethics Statement

The studies involving human participants were reviewed and approved by the Institutional Review Board (IRB) of Kaohsiung Medical University Hospital (IRB Number: KMUH-IRB-20140303). The patients/participants provided their written informed consent to participate in this study.

## Author Contributions

C-YL: Conception and design, acquisition of data, analysis and interpretation of data, drafting of the manuscript, final approval of the manuscript, statistical analysis, literature research, clinical studies, experimental studies, and obtaining funding. C-HH: Data acquisition, analysis and interpretation of data, drafting of the manuscript, final approval of the manuscript, literature research, clinical studies, and experimental studies. W-HW: Data acquisition, analysis and interpretation of data, drafting of the manuscript, final approval of the manuscript, and experimental studies. JT: Analysis and interpretation of data, critical revision of the manuscript, final approval of the manuscript, administrative, technical, or material support, and supervision. L-CH: Data acquisition, drafting of the manuscript, final approval of the manuscript, administrative, and technical or material support. C-CL: Data acquisition, drafting of the manuscript, and final approval of the manuscript. Y-CC: Data acquisition, drafting of the manuscript, and final approval of the manuscript. Y-HC: Conception and design, acquisition of data, analysis and interpretation of data, critical revision of the manuscript, final approval of the manuscript, acting as a guarantor of integrity of the entire study, statistical analysis, clinical studies, obtaining funding, and supervision. W-TL: Conception and design, acquisition of data, analysis and interpretation of data, critical revision of the manuscript, final approval of the manuscript, acting as a guarantor of integrity of the entire study, statistical analysis, literature research, experimental studies, obtaining funding, and supervision. All authors contributed to the article and approved the submitted version.

## Funding

This study was supported by grants from the Ministry of Science and Technology (MOST103-2314-B-037-068; MOST104-2314-B-037-079; MOST106-2320-B-037-030-MY3; MOST107-2314-B-037-087; MOST108-2314-B-037-046-), Kaohsiung Medical University (KMU-TP103A13; KMU-TP104A30; KMU-TC109A01-1), and Kaohsiung Medical University Hospital (KMUH104-4R14).

## Conflict of Interest

The authors declare that the research was conducted in the absence of any commercial or financial relationships that could be construed as a potential conflict of interest.
