# Supplementary material for: Mono-(2-ethylhexyl) phthalate Promotes Dengue Virus Infection by Decreasing IL-23-Mediated Antiviral Responses
Source: Front Immunol. 2021 Feb 15;12:599345. doi: 10.3389/fimmu.2021.599345 (PMC7919524; doi:10.3389/fimmu.2021.599345)
Supplement: Supplementary file 1 [file DataSheet_1.pdf]

Supplementary Data

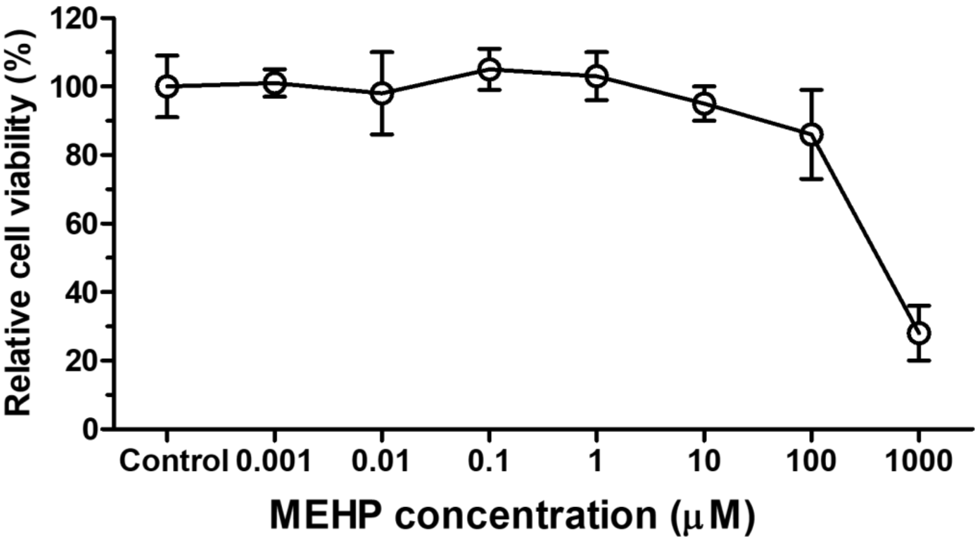

Supplementary Figure S1. MEHP cytotoxicity test. Human MDMs (10^6/ml) were cultured in RPMI 1640 medium with 10% FBS and treated with 1 nM to 1 mM of MEHP for 48 hours. After treatments, MDM viability was tested by XTT cytotoxicity assay. MEHP lower than 0.1 mM did not show cytotoxic effects, and the ID50 of MEHP is about 0.5 mM. *P<0.05; MEHP-treated vs. control; analyzed by the Kruskal-Wallis test; N=3; means ± SDs

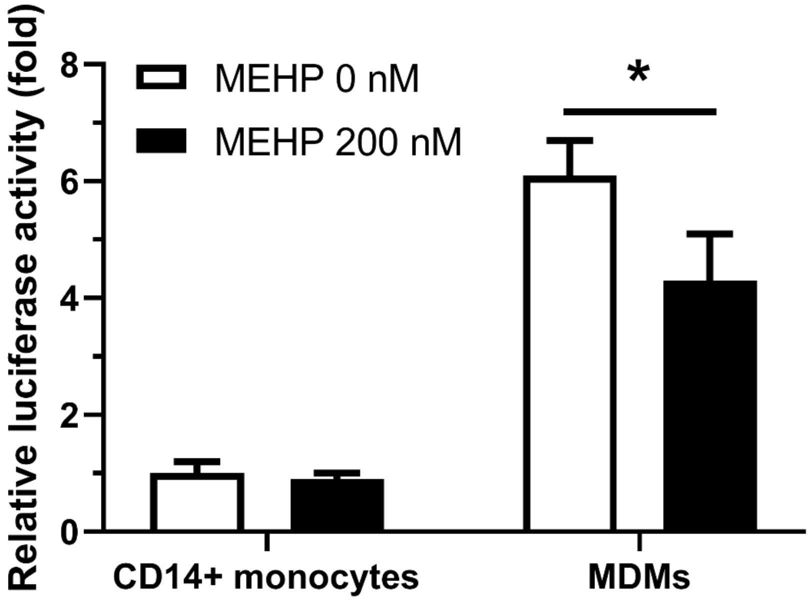

Supplementary Figure S2. Luciferase activity assays with recombinase PPAR-responding element promoters were assayed in CD14+ cells and monocyte-derived macrophages (MDMs). MEHP treatment significantly reduced the activity of PPARγ. *P < 0.05 by Kruskal-Wallis test; N = 6, means ± SD.
