# Supplementary material for: Mono-(2-ethylhexyl) phthalate Promotes Dengue Virus Infection by Decreasing IL-23-Mediated Antiviral Responses
Source: Front Immunol. 2021 Feb 15;12:599345. doi: 10.3389/fimmu.2021.599345 (PMC7919524; doi:10.3389/fimmu.2021.599345)
Supplement: Supplementary file 2 [file DataSheet_2.docx]

**Supplementary Table S1**

| Primers and probes used in this study. | | |
| --- | --- | --- |
| **For dengue virus RNA detection by RT-PCR** | |  |
| DENV1-F | 5’-CAAAAGGAAGTCGYGCAATA-3’ |  |
| DENV1-R | 5’-CTGAGTGAATTCTCTCTGCTRAAC-3’ |  |
| DENV1-P | 5’-CATGTGGYTGGGAGCRCGC-3’ |  |
| DENV2-F | 5’-CAGGCTATGGCACYGTCACGAT-3’ |  |
| DENV2-R | 5’-CCATYTGCAGCARCACCATCTC-3’ |  |
| DENV2-P | 5’-CTCYCCRAGAACGGGCCTCGACTTCAA-3’ |  |
| DENV3-F | 5’-GGACTRGACACACGCACCCA-3’ |  |
| DENV3-R | 5’-CATGTCTCTACCTTCTCGACTTGYCT-3’ |  |
| DENV3-P | 5’-ACCTGGATGTCGGCTGAAGGAGCTTG-3’ |  |
| DENV4-F | 5’-TTGTCCTAATGATGCTRGTCG-3’ |  |
| DENV4-R | 5’-TCCACCYGAGACTCCTTCCA-3’ |  |
| DENV4-P | 5’-TYCCTACYCCTACGCATCGCATTCCG-3’ |  |

**Supplementary Table S2**


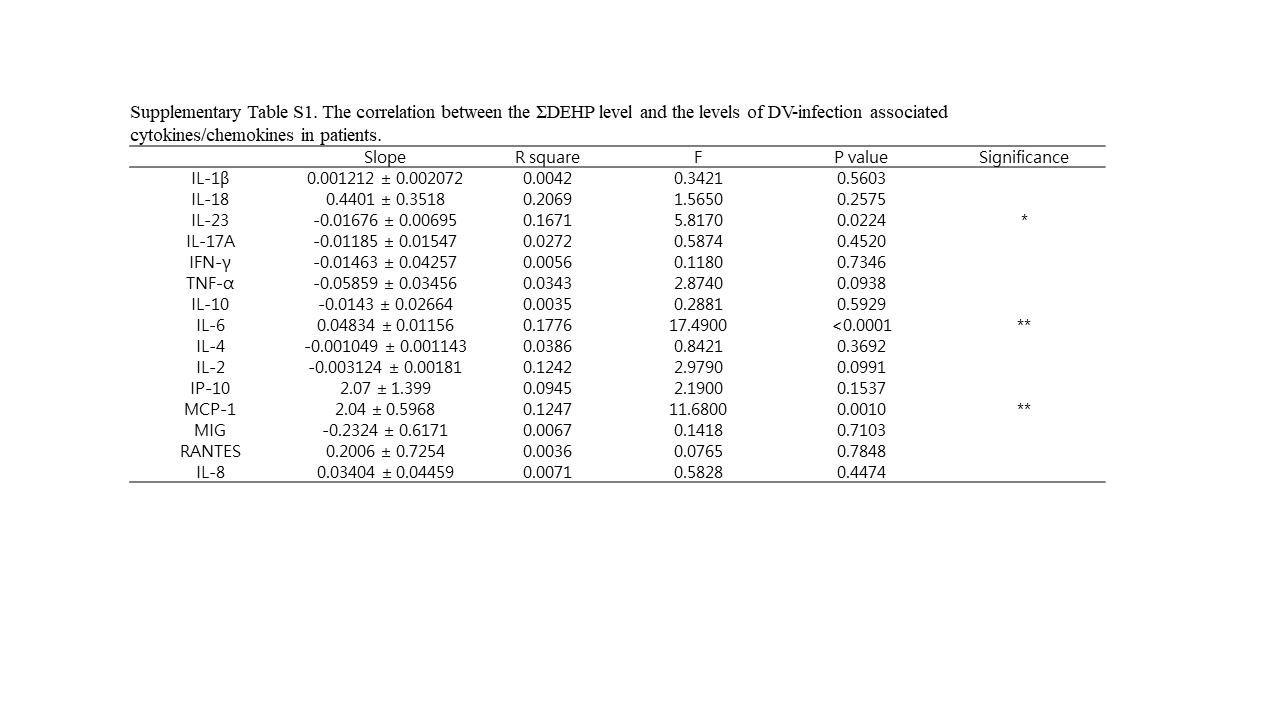
Correlations between ΣDEHP level and levels of DV-infection-associated cytokines/chemokines in patients with dengue fever.

*P<0.05; **P<0.01, analyzed by linear regression

**Supplementary Table S3**


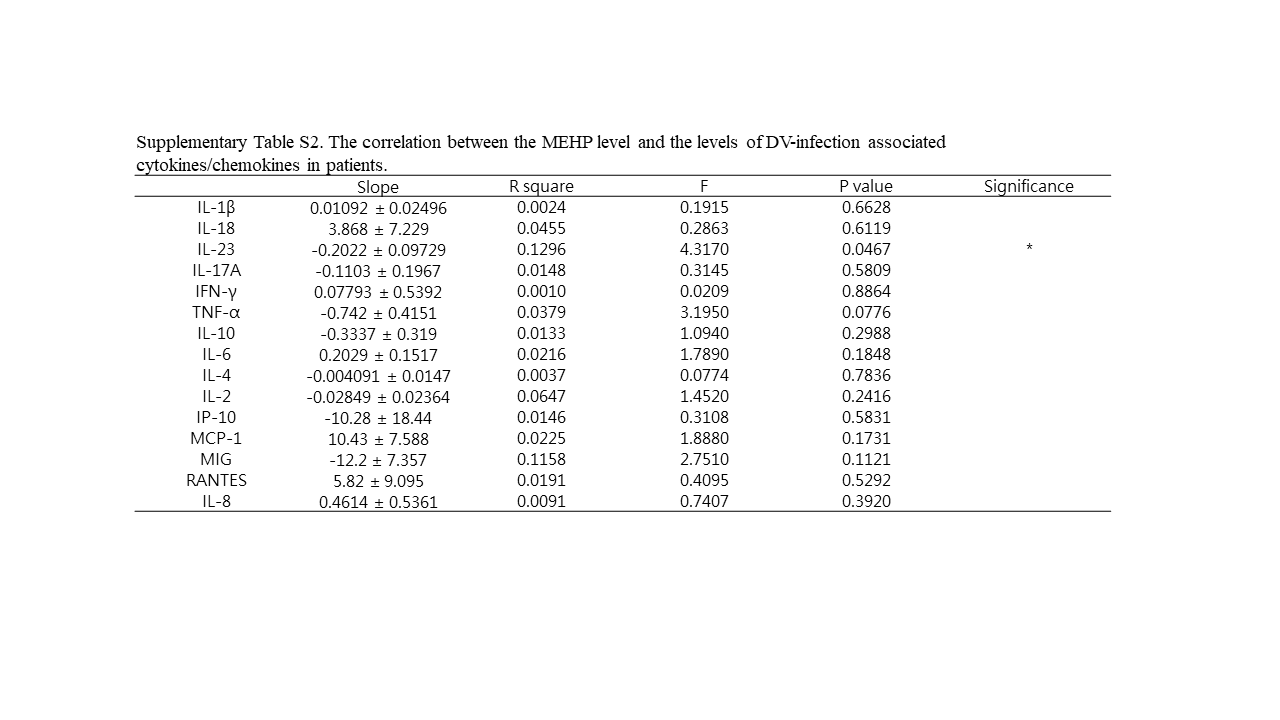
 Correlations between MEHP level and levels of DV-infection-associated cytokines/chemokines in patients with dengue fever.

*P<0.05; **P<0.01, analyzed by linear regression

**Supplementary Table S4**


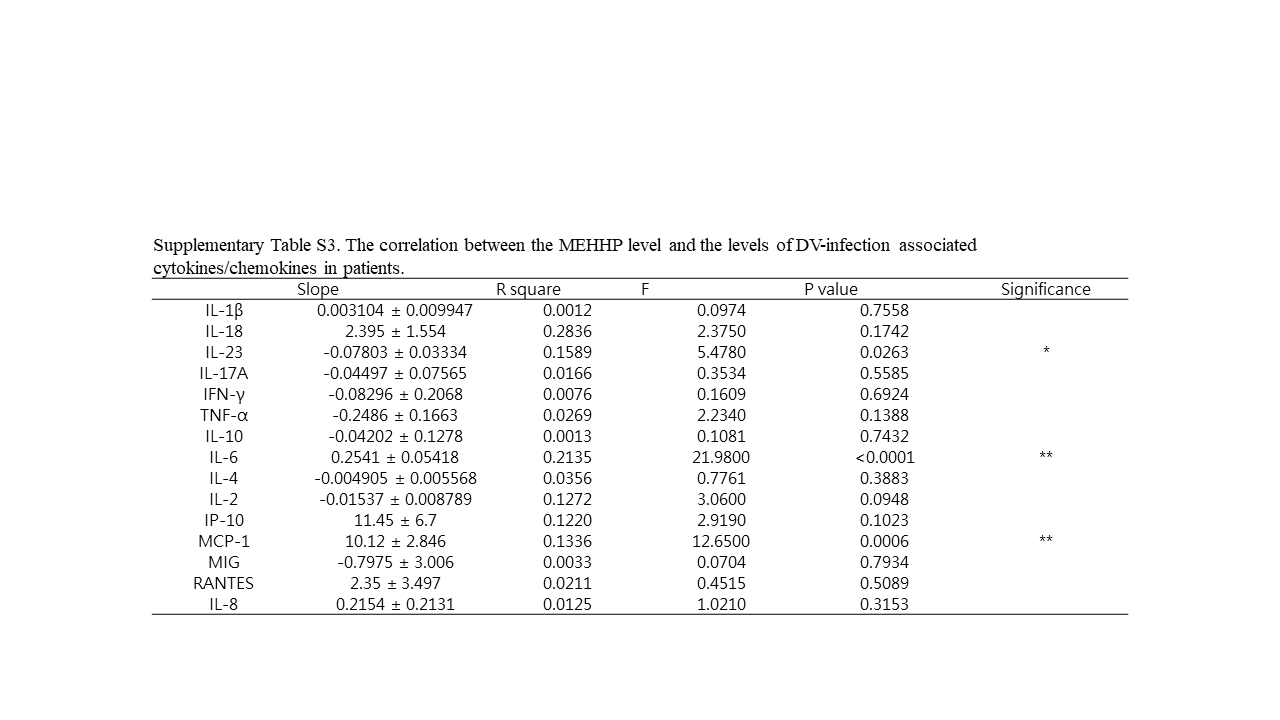
 Correlations between MEHHP level and levels of DV-infection-associated cytokines/chemokines in patients with dengue fever.

*P<0.05; **P<0.01, analyzed by linear regression

**Supplementary Table S5**


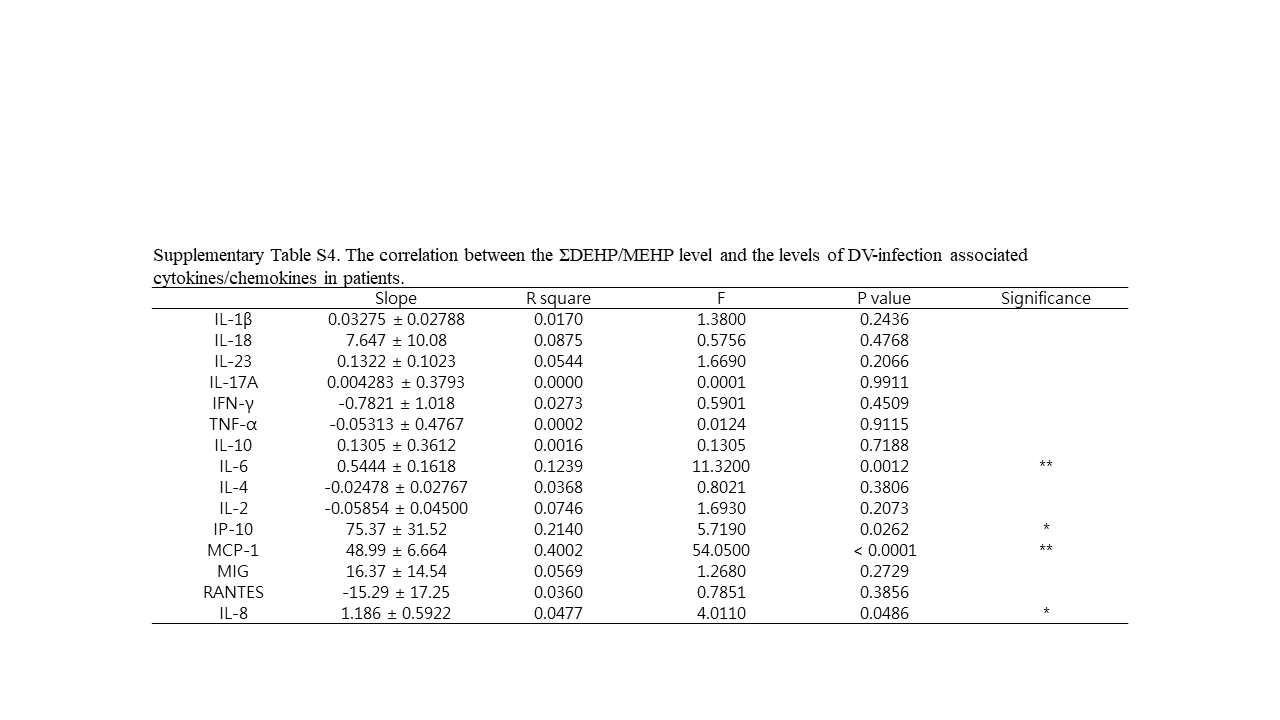
 Correlations between ΣDEHP/MEHP level and levels of DV-infection-associated cytokines/chemokines in patients with dengue fever.

*P<0.05; **P<0.01, analyzed by linear regression

**Supplementary Table S6**


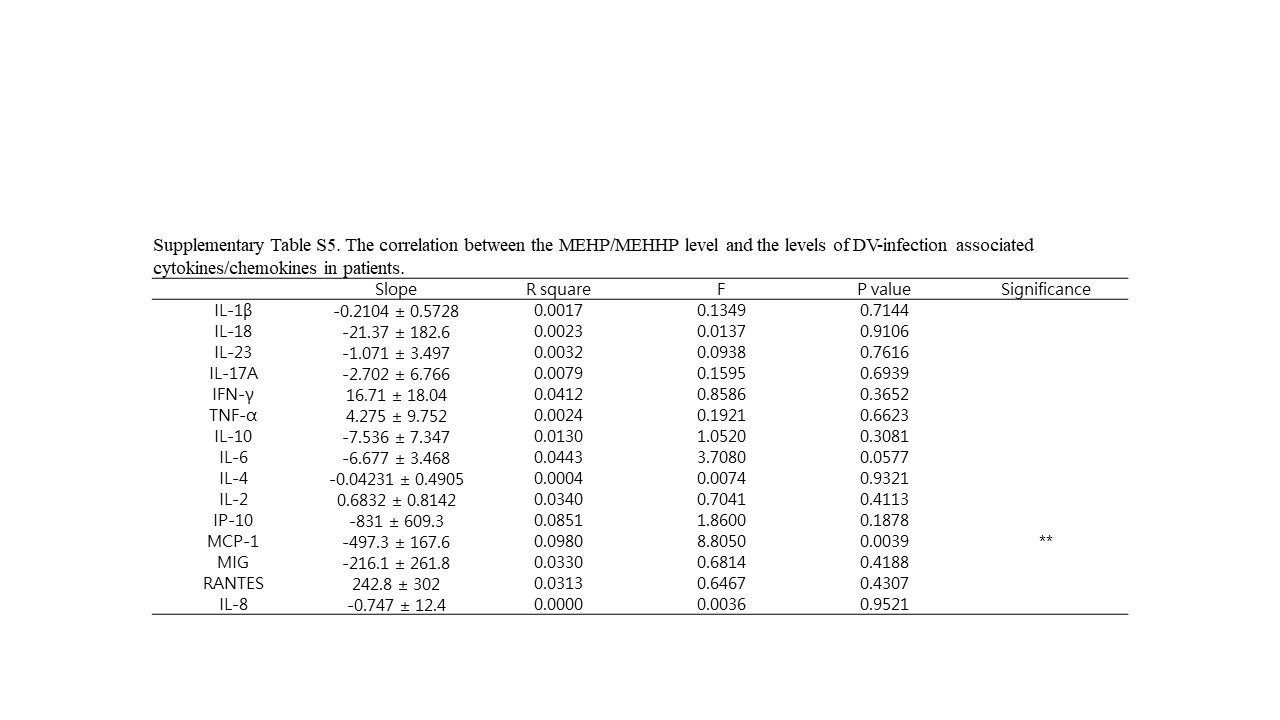
 Correlations between MEHP/MEHHP level and levels of DV-infection-associated cytokines/chemokines in patients with dengue fever.

*P<0.05; **P<0.01, analyzed by linear regression
